# Glaucoma Community Care: Does Ongoing Shared Care Work?

**DOI:** 10.5334/ijic.5470

**Published:** 2020-08-07

**Authors:** Angelica Ly, Elizabeth Wong, Jessie Huang, Michael Yapp, Katherine Masselos, Michael Hennessy, Michael Kalloniatis, Barbara Zangerl

**Affiliations:** 1 Centre for Eye Health, Sydney, NSW, AU; 2 School of Optometry and Vision Science, UNSW Sydney, Sydney, NSW, AU; 3 Department of Ophthalmology, Prince of Wales Hospital, Sydney, NSW, AU

**Keywords:** collaborative care, chronic eye disease, telemedicine, task-shifting

## Abstract

**Purpose:**

We assessed a novel, public, vertical integrated care model for glaucoma management in the community.

**Methods:**

This study was a retrospective, longitudinal study of 266 patients diagnosed or suspected of glaucoma. Patients were stratified to either ongoing ophthalmology-led (n = 81) or optometry-led shared care (n = 185). Demographics and clinical characteristics, including the re-referral rate and timeliness of follow up were analysed.

**Results:**

Just under half (565/1224, 46%) of all follow up consultations over the total study period of 45 months were seen in optometry-led care, with a re-referral rate to ophthalmology of 21%. Treated patients showed a median intraocular pressure reduction of 20% and a median time delay of just two days between the actual and recommended review period.

**Conclusions:**

Shared care provides an effective option for managing the ongoing care burden in chronic stable glaucoma cases at low risk of vision loss.

## Introduction

Glaucoma is a progressive optic neuropathy and a leading cause of vision impairment worldwide that requires life-long surveillance and management [1]. Health care professionals, including optometrists, are typically involved in case finding and refer to specialists (ophthalmologists) for treatment. However, ophthalmology workforce numbers, limited health care resources, and the ageing population have led to unacceptable care delays and associated vision loss among patients accessing public health care [2,3]. In Australia, there are only 990 qualified ophthalmologists to meet the demand and only 16 per cent are employed in the public sector [4]. These ophthalmology workforce numbers are not expected to grow in line with the expansion of the aged population [5], which has thus led to a growing global interest in glaucoma shared care models [1,6,7,8,9,10,11,12,13].

Shared care occurs when patient care is “provided by two or more health practitioners, each practising in their sphere of expertise in consultation with the patient” and may range from ad hoc to formal agreements varying according to contextual factors such as resources, urban versus regional settings, remuneration, training, equipment, scope of practice, responsibilities and practitioner skill [9,12,14]. In glaucoma, such schemes typically aim to better utilise health care resources through the vertical integration of public ophthalmology departments and community optometrists [13]. Optometrists with specific training reach high degrees of agreement with ophthalmologists in clinical decision making [7,15,16,17]. Suitably trained optometrists have also shown good adherence to guidelines regarding initial treatment decisions and the timing of regular monitoring [8,18]. Yet, there is a paucity of data on successful glaucoma shared care schemes outside of the United Kingdom [1].

Guidelines [19] state that the aims of shared care in glaucoma are to provide patient-centred, evidence-based, accessible care that minimises unnecessary treatment. In previous work, we described the value of optometry-ophthalmology shared care in referral refinement [20] and the baseline characteristics of patients entering into a hybrid care model [10]. However, a demonstration of the feasibility and longitudinal impact on patient outcomes is needed. In this study, we describe the activity and effectiveness of a protocol-based, virtual review-facilitated, glaucoma vertical integrated care model operating in Sydney, Australia using care outcomes including intraocular pressure, progression in visual fields mean deviation, and adherence to follow up.

## Methods

The shared care scheme described herein is a joint initiative of Guide Dogs NSW/ACT, University of New South Wales Sydney and the Prince of Wales hospital ophthalmology department. It was designed in accordance with local guidelines [14,19,21,22] for the ongoing treatment and management of early to moderate glaucoma. Consultant ophthalmologists from Prince of Wales hospital (MPH and KM) are involved in the ongoing governance, training of participating staff, development of clinical protocols and quality assurance. The scheme aims to share the burden of ongoing glaucoma care across both clinical settings and professional groups. It represents a hybrid of a previously described optometry-led clinic for referral refinement [20] (also known as an intermediate-tier care model [23]), and an ophthalmology-led glaucoma management clinic [10].

Criteria for entry into the ophthalmology-led glaucoma management clinic are provided as supplementary material and have been published previously [10]. Patients are stratified according to disease severity and stability, and a follow up consultation is subsequently arranged either in the glaucoma management clinic (if face-to-face ophthalmological opinion is required) or in the optometry-led clinic [20,23] (involving a technician and a highly-trained optometrist, without an ophthalmologist). In the glaucoma management clinic, optometrists facilitate and confer on ongoing care but ophthalmologists lead the clinical decision making. Stable or low risk cases are shifted into optometry-led shared care whereby the case notes, imaging findings and report are reviewed virtually (typically on a different day) by either a senior peer optometrist or consultant ophthalmologist remotely; there is no face to face consultation between the patient and the reviewing clinician. In most instances in which treatment is initiated or altered by an ophthalmologist in the glaucoma management clinic, a short subsequent consultation involving a review of symptoms and intraocular pressure (without any additional imaging and/or perimetry) follows four to eight weeks later. In the current model, this appointment is conducted by an optometrist only (optometry-led) and occurs when concurrent face-to-face ophthalmological assessment is available if required.

### Inclusion criteria

A retrospective record review of all patients seen in the ophthalmology-led glaucoma management clinic was conducted. For this study, patients were stratified into two groups. The ophthalmology-led care group denotes patients seen in the glaucoma management clinic only (including short subsequent consultations) over the total study period. Patients were alternatively categorised into the shared care group if at least one of their follow up consultations over the total study period was performed in the optometry-led clinic. To be eligible, all patients underwent a baseline comprehensive assessment in the glaucoma management clinic between the 18/03/2015 and 14/03/2018 and were seen at least once for a follow up consultation in the period ending on the 31/12/2018. All subjects provided informed written consent in accordance with the Declaration of Helsinki, approved by a Biomedical Human Research Ethics Advisory Panel, University of New South Wales Sydney.

### Data collection

Patient demographic and clinical data were extracted from the patient’s medical record (VIP.net, Best Practice Software, Bundaberg, QLD, Australia). The Humphrey visual fields mean deviation progression rate (a global measure of the patient’s overall deterioration in visual function relevant to glaucoma) was extracted from the instrument software (Forum Viewer Version: 4.2.1.66, Carl Zeiss Meditec, Dublin, California, USA) where available, using all available historical data, up until 10/05/2019. One patient with a mean deviation positive progression rate of +7.4dB/year was excluded from the analysis. A patient with a progression rate worse than –1dB/year was considered a “fast progressor” and the total number of patients with a negative slope of mean deviation values (indicating any progression) was also identified [24]. Further details on the study coding protocol are provided in supplementary file 2.

Study outcome measures included: 1) clinical characteristics, 2) total number, types of consultations and re-referral rate over time, 3) clinical management and recommended review period for each consultation, and 4) care effectiveness defined using adherence to follow up, intraocular pressure and visual fields mean deviation progression. Cases where a scheduled follow up appointment within the study period was missed were determined using the patient’s final visit within the study period crosschecked against the associated report recommendation where applicable (n = 230). One count was assigned for each occurrence where the recommended recall date was earlier than the 30/11/2018.

### Statistical analysis

All statistical analyses were performed using software package SPSS (version 25, IBM corporation, Chicago, USA). Figures were generated using GraphPad Prism (version 7, GraphPad software, California, USA). Coded data were analysed using frequencies of occurrence. Chi-square, Fisher’s exact or the Mann Whitney U-test was used to identify statistically significant differences between groups. All statistical tests were performed two-sided and at a 5% significance level.

## Results

A total of 1,490 medical records from 266 principally Caucasian (56%) patients, 170 males (64%) and 96 females (36%) ranging in age from 23 to 86 years of age (Table 1), met the inclusion criteria of the study. Patients were followed a median of five times (range of 2 to 14) totalling 1,224 follow up consultations over the study period of three years, representing a 460% increase in raw consultation numbers. The baseline diagnosis was most commonly open angle glaucoma (140, 53%) followed by glaucoma suspect (91, 34%) and ocular hypertension (19, 7%). Fourteen patients (5%) had secondary open angle glaucoma, including pigment dispersion or pseudoexfoliation. Two patients (1%) had other forms of glaucoma at baseline.

**Table 1:** Demographic details of the study patients.

Characteristic	Total sample (n = 266)	Ophthalmology-led care group (n = 81)	Shared care group (n = 185)	P-value

Age, years				
Mean (SD)	62 (12)	60 (13)	62 (12)	0.276
Range	23–86	23–86	25–86	
Sex, n (%)				
Male	170 (64%)	53 (65%)	117 (63%)	0.782
Female	96 (36%)	28 (35%)	68 (37%)	
Ethnicity, n (%)				
Caucasian	150 (56%)	46 (57%)	104 (56%)	0.894
Asian	83 (31%)	24 (30%)	59 (32%)	
Other^†^	33 (12%)	11 (14%)	22 (12%)	
Baseline refraction^‡^				
Spherical equivalent, mean (SD)	–0.7 (2.5)	–1.0 (2.7)	–0.6 (2.5)	0.742
Myopia < –1, n (%)	87 (34%)	24 (32%)	63 (35%)	0.667
Myopia < –3, n (%)	42 (16%)	16 (21%)	26 (14%)	0.196
Baseline maximum IOP in both eyes, mmHg				
Mean (SD)	19 (5)	20 (5)	19 (5)	0.070
Range	8–34	10–34	8–33	
IOP ≥ 22	78 (29%)	30 (37%)	48 (26%)	0.079
IOP < 22	188 (71%)	51 (63%)	137 (74%)	
Baseline thinnest CCT in both eyes, µm				
Mean (SD)	548 (34)	547 (36)	549 (33)	0.381
Range	452–656	452–635	454–656	
CCT < 555	151 (57%)	48 (59%)	103 (56%)	0.687
CCT ≥ 555	115 (43%)	33 (41%)	82 (44%)	
Baseline glaucoma severity^§^				
Suspect or OHT	110 (43%)	20 (26%)	90 (51%)	<0.001***
Early	89 (35%)	22 (29%)	67 (38%)	
Moderate	10 (4%)	6 (8%)	4 (2%)	
Advanced	46 (18%)	29 (38%)	17 (10%)	
Primary prescribed therapy, n (%)				
Prostaglandin analogue	112 (68%)	38 (61%)	74 (72%)	0.475
Laser (SLT or PI)	23 (14%)	9 (15%)	14 (14%)	
Alpha-agonist	3 (2%)	1 (2%)	2 (2%)	
Beta-blocker	3 (2%)	1 (2%)	2 (2%)	
Combination	24 (15%)	13 (21%)	11 (11%)	
Initial recommended review period^¶^				
<3 months	108 (47%)	38 (54%)	70 (44%)	0.061
3–5 months	30 (13%)	13 (19%)	17 (11%)	
6–8 months	83 (36%)	17 (24%)	66 (42%)	
9–12 months	7 (3%)	2 (3%)	5 (3%)	

^†^ Includes four individuals of African descent. ^‡^ Missing values: 11. ^§^ Missing values: 12. ^¶^ Missing values: 38; Advanced glaucoma was defined as a visual field mean deviation worse than –12dB, or three or more points with an abnormal probability score of less than 2% within the central ten degrees. Further data on the severity of visual field defects using mean deviation alone is presented in Figure 2D. Abbreviations: CCT, central corneal thickness; IOP, intraocular pressure; OHT, ocular hypertension; PI, peripheral iridotomy; SD, standard deviation; SLT, selective laser trabeculoplasty.

**Figure 1:**
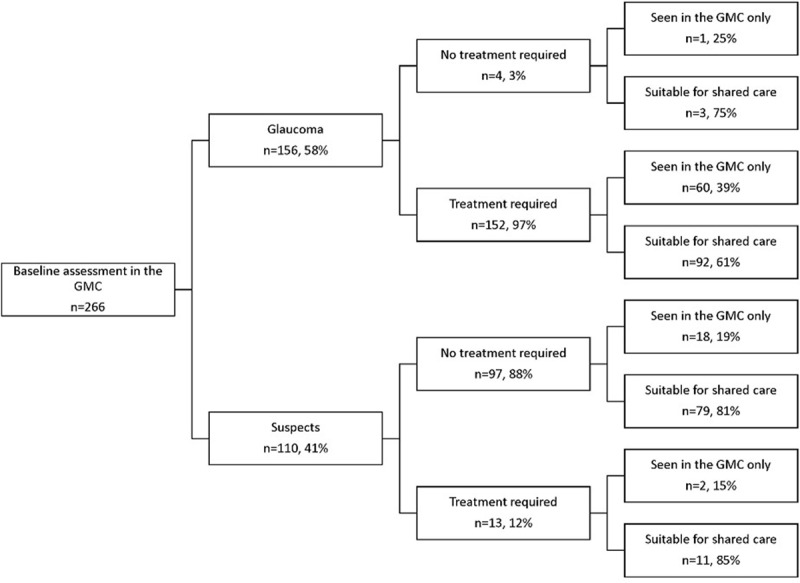
Distribution of all patients enrolled in the scheme. Patients were categorised as suitable for shared care if at least one of their follow up consultations over the three-year study period was conducted in the optometry-led clinic. Abbreviations: GMC, glaucoma management clinic.

Treatment was typically initiated, maintained or changed for the majority of patients diagnosed with glaucoma at baseline (152, 97%; Figure 1). There were four instances (3%) where treatment was postponed pending further investigation (phasing of applanation intraocular pressure or neuroimaging). Similarly, most glaucoma suspects or patients with ocular hypertension did not require treatment (97, 88%). There were thirteen cases (12%) where treatment was required, typically due to associated risk factors, including occludable angles, pigment dispersion syndrome, pseudoexfoliation signs or Drance haemorrhage. Three cases were advised to continue their prior glaucoma treatment plan.

### Clinical characteristics of patients suitable for shared care

**Figure 2:**
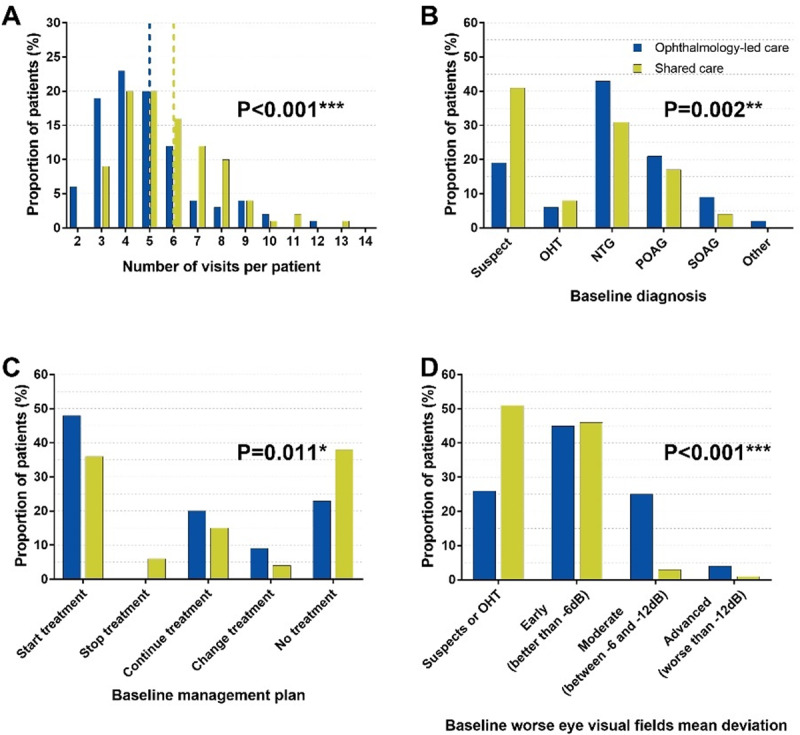
Key differences between the groups of patients suitable and unsuitable for shared care: **A)** The blue and green vertical dotted lines indicate the median of 5 and 6 visits for the ophthalmology-led care only and shared care groups, respectively. **B)** Baseline diagnosis and **C)** Management across the two groups **D)** Patients in ophthalmology-led care showed a poorer worse eye Humphrey visual fields mean deviation median (interquartile range) of –3.70 (5.19) dB compared to –1.58 (3.18) dB for the shared care group. Abbreviations: dB, decibels; NTG, normal tension glaucoma; OHT, ocular hypertension; POAG, primary open angle glaucoma; SOAG, secondary open angle glaucoma.

Patients enrolled in the study were mostly suitable for shared care (185, 70%). Considering attendances per patient, patients suitable for shared care were seen more often over the total study period (Figure 2A). They were statistically more likely to have a baseline diagnosis of glaucoma suspect rather than glaucoma (Figure 2B). They were also less likely to be treated (Figure 2C) and typically did not have a moderate or advanced visual field defect (Figure 2D). There was a trend toward higher intraocular pressure and shorter review periods in the ophthalmology-led care group (implying reduced disease stability); however, these differences were not statistically significant. There was no statistically significant difference in age, sex, ethnicity, baseline refraction, central corneal thickness, or the prescribed therapy between patients suitable versus non-suitable for shared care (Table 1).

### Patients’ journey of care

**Figure 3:**
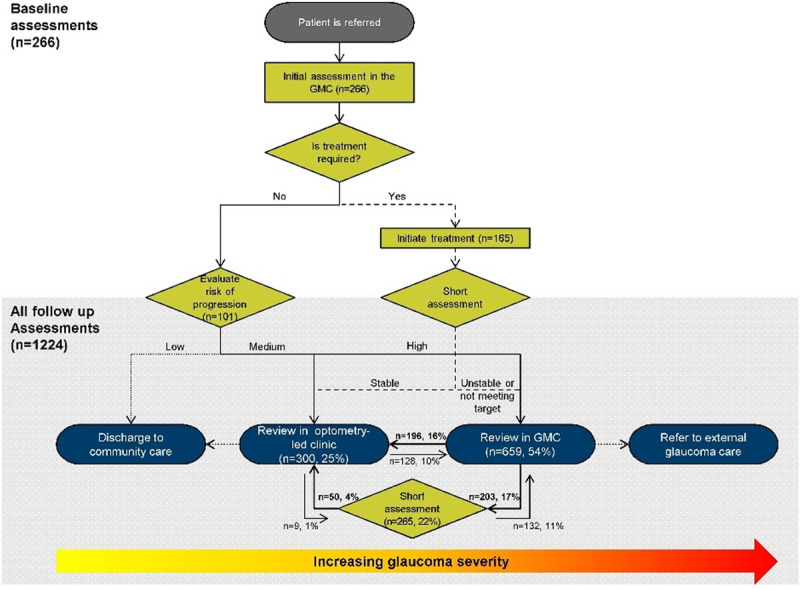
Flowchart illustrating the direction of patient flow across all 1,490 attendances required for the 266 patients included in the study. Almost half (46%) of all follow up assessments were seen by optometrists only; either in optometry-led clinic or as a short subsequent consultation. Based on follow up attendances only, the re-referral rate to ophthalmology in the GMC was 21% (260 attendances). Abbreviations: GMC, glaucoma management clinic.

Just under half (565, 46%) of all follow up consultations were conducted in optometry-led care: one quarter (300, 25%) were seen in the optometry-led clinic and the remainder (265, 22%) were short subsequent consultations. The outstanding numbers of follow up consultations (659, 54%) were seen in the ophthalmology-led glaucoma management clinic (Figure 3). Considering follow up attendances only, glaucoma management clinic visits were typically followed by another glaucoma management clinic visit (399/1,224, 33%) due to a relatively less stable or higher risk presentation, followed with a short subsequent consultation (203/1,224, 17%) or transferred directly into the optometry-led clinic (196/1,224, 16%). Patients were re-referred from the optometry-led clinic into the ophthalmology-led glaucoma management clinic in 128 instances (10%). This direction of patient flow differed significantly between the cases not requiring treatment versus those in which treatment was initiated, changed or continued (Chi-square p < 0.001).

The relationship between diagnosis, management and the recommended review period is outlined in Table 2. Instances where therapy was initiated, changed or discontinued were typically followed closely (<3 months). Variation within a row reflects management titration according to risk of progression consistent with national guidelines [22], for example, in the group of glaucoma suspects not receiving any treatment, the majority (137/247, 55%) were reviewed in 6–8 months, 60 (24%) were reviewed in 9–12 months, while the outstanding number (50/249, 20%) were reviewed more closely (<6 months). There were 834 (62%) attendances where the case was deemed stable (based on a recommended review period greater than or equal to six months).

**Table 2:** Cross tabulation of the diagnosis versus action plan across all appointments where a specific review period was applicable (n = 1,348).

Diagnosis	Treated	Management plan	Recommended review period				

			<3 months	3–5 months	6–8 months	9–12 months	Total

Suspect	Untreated	Stop treatment	10	3	1	0	14
		No treatment	9	41	137	60	247
	Treated	Start treatment	1	0	0	0	1
		Continue treatment	0	2	17	3	22
OHT	Untreated	Stop treatment	1	1	0	0	2
		No treatment	3	16	52	13	84
	Treated	Start treatment	4	0	2	0	6
		Continue treatment	1	5	24	0	30
		Change treatment	2	0	0	0	2
NTG	Untreated	No treatment	1	0	0	0	1
	Treated	Start treatment	49	2	1	0	52
		Continue treatment	19	94	310	3	426
		Change treatment	45	9	5	0	59
POAG	Untreated	Stop treatment	1	1	0	0	2
		No treatment	1	2	0	0	3
	Treated	Start treatment	37	2	0	0	39
		Continue treatment	17	63	158	1	239
		Change treatment	37	4	5	0	46
SOAG	Treated	Start treatment	5	1	0	0	6
		Continue treatment	5	14	37	0	56
		Change treatment	8	1	2	0	11
							1348

Abbreviations: NTG, normal tension glaucoma; OHT, ocular hypertension; POAG, primary open angle glaucoma; SOAG, secondary open angle glaucoma.

There were 64 instances where the recommended review period was contingent on some external variable (usually hospital scheduling for selective laser trabeculoplasty, peripheral iridotomy, neuroimaging, repeat visual fields findings or intraocular pressure phasing in primary care). There were 28 instances where the patient was referred from the scheme into glaucoma ophthalmological care elsewhere, which could occur at any visit during the study period. This ranged from the first to the eighth consecutive visit (median of 3) and occurred most commonly due to an advanced visual field defect (15, 54%), pre-existing ophthalmological care (5, 18%), monocular status (2, 7%), or a history of glaucoma related ocular surgery (2, 7%). Two patients were discharged back into primary care because of confirmed low risk glaucoma status, and a similar minority (2 cases, 4%) elected to leave the scheme because they were moving overseas.

### Effectiveness of the shared care scheme

There were 42/230 (18%) cases with a recommended recall date on or before the 30/11/2018, where a follow up consultation within the study period was missed. In ten cases, there were reasons documented in the medical record, for example, the patients had deceased, or elected care closer to home. Thirteen cases were resolved by reviewing the patients’ medical records at the time of writing i.e. the follow up consultation was delayed but occurred at a time outside of the study period. Nineteen cases (8% over three years) were lost to follow up with reasons unknown. The proportion of cases with a missed follow up appointment within the study period was also significantly higher in the ophthalmology-led care group (17/55, 31%), compared to the group of patients undergoing shared care (25/175, 14%).

**Figure 4:**
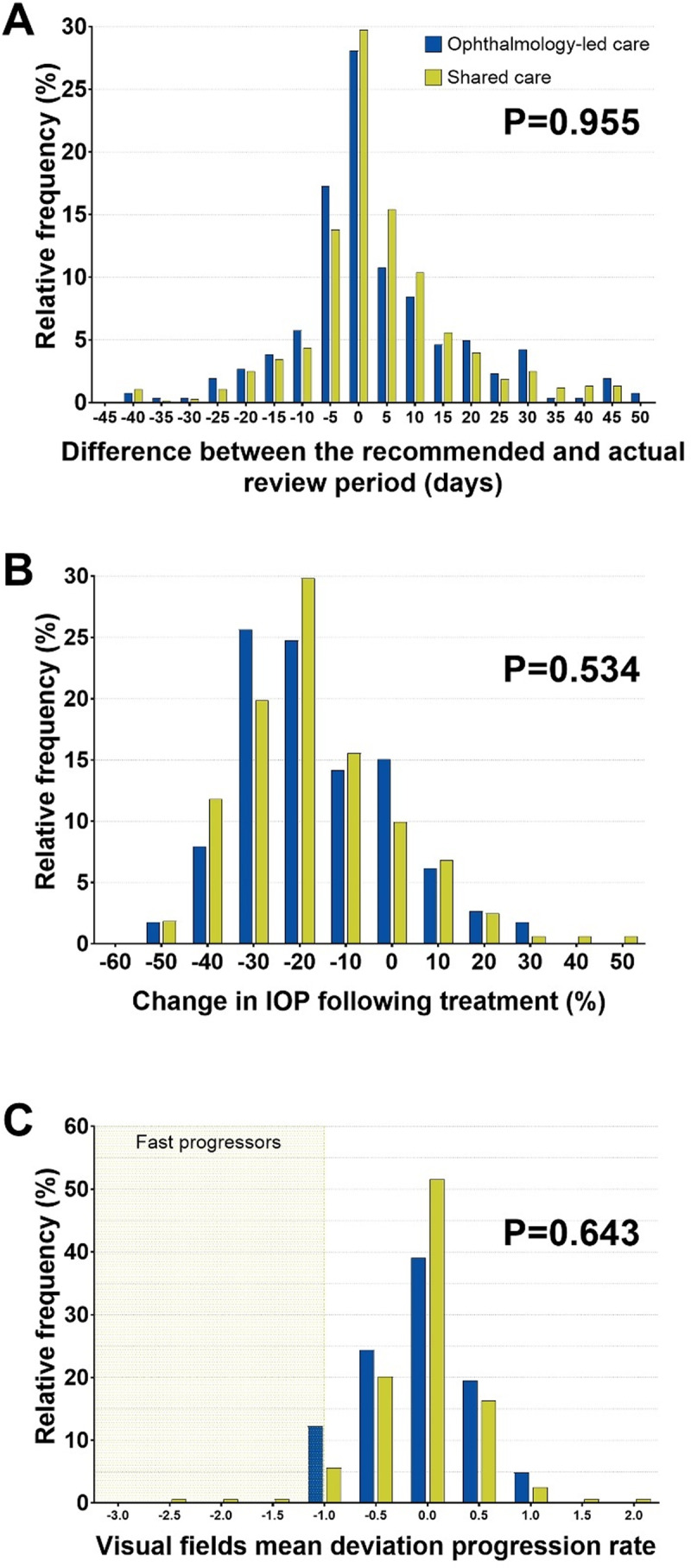
Frequency histograms for the key outcome measures of the study: there was no statistically significant difference between the ophthalmology-led care and shared care groups in **A)** timeliness of follow up appointments, after removing outliers, **B)** change in IOP with treatment, or **C)** visual fields mean deviation progression rates among the worse eye. Shaded in green are 7 subjects that showed a fast visual fields mean deviation progression rate (worse than –1dB/year). Abbreviations: IOP, intraocular pressure.

Other care effectiveness measures, including the timeliness of follow up (Figure 4A), and change in intraocular pressure with treatment (Figure 4B), did not differ significantly between both groups. Consistent with the data presented in Table 1, cases with more severe visual field defects at their final follow up visit were seen in ophthalmology-led care only (Chi-square p = 0.001). Approximately half (101/200; 51%) of all patients demonstrated some progression; however, a minority (7 patients, 3.5%) progressed at a rate worse than –1dB/year (Figure 4C). Notably, 72 patients (36%) displayed a positive rate of mean deviation change, which likely reflects a combination of high visual field variability and learning effect. The distribution of visual field severity did not change significantly between baseline and follow up in either the shared care (Chi-square p = 0.6740) or ophthalmology-led care group (Chi-square, p = 0.2184).

## Discussion

These results add to the growing body of evidence showing that a team approach provides an effective solution for managing the ongoing care burden in chronic stable glaucoma cases at low risk of vision loss for the benefit of patient outcomes [6,7,25]. Contrary to other vertical integrated care schemes, which typically shift pre-existing, stable patients from a public hospital outpatients department to a community care model, patients in this scheme were identified and referred directly by primary care providers. Thus, patients avoided entry into the public hospital clinical service unless laser, surgical treatment or close monitoring and treatment of advanced glaucoma was required. The study also confirms that the service is appropriately targeted; a 59% majority of patients entering into the scheme had glaucoma at baseline and 33% of glaucoma suspects, and 18% of glaucoma patients may not be otherwise receiving appropriate follow up [20].

Just under half of the ongoing care burden for patients seen in this Sydney shared care model was shifted successfully to an optometry workforce. This has possible beneficial implications in reducing the long wait times for non-urgent, specialist care in the public health system in Australia and other countries that seek to provide universal health care [26]. Re-referral to the glaucoma management clinic for a specialists’ opinion occurred less often (260/1,224, 21%) at a level similar to previous studies (ranging from 13.2% to 55%) [1,7,9]. Shared care patients also experienced an improvement in loss to follow up and a median time delay of just two days between the actual and recommended review period. The majority (87%) of all consultations were also seen within one month of the recommended time frame. This is valuable because fewer and shorter delays in care translate to a lower incidence of overall disease progression, and thus better patient outcomes [7].

In this scheme, patients were allocated to either ongoing ophthalmology-led or optometry-led care based on the consensus decision of the examining optometrist and ophthalmologist. The short subsequent consultations following initiation of treatment provided the primary mechanism of transitioning patients safely between the two, which was only possible due to the high level of training (including therapeutic endorsement [27]) of the participating optometrists. The importance of additional training in areas of interest has been emphasised previously in Ireland and Canada [28,29]. Allocation decisions were driven by factors described in the peer-reviewed literature including: loss of visual acuity, intraocular pressure exceeding target, signs suspicious of structural or functional progression (such as a new disc haemorrhage, retinal nerve fibre layer defect, change in the disc rim), and/or intolerance to treatment [1,6,11]. Patients initiated on therapy and subsequently reviewed 6–8 weeks later for a short subsequent consultation might have extra tests performed and interpreted at the discretion of the examining optometrist e.g. if baseline visual fields were unreliable, or to monitor the resolution of a Drance haemorrhage. The examining optometrist could then exercise their independent clinical decision making to manage the patient and determine whether additional virtual or face-to-face ophthalmological opinion was also required. This was especially vital in cases where the review period was protracted, e.g. if contingent on hospital scheduling for selective laser trabeculoplasty or external neuroimaging.

### Potential for expansion of the current practice model

In Australia, optometrists provide over 75 per cent of vision care services [30]. They are skilled in the assessment, detection and management of ocular disease, and a majority (58.2 per cent) also hold therapeutic endorsement. Since 2008, optometrists have been able to independently initiate topical therapy for glaucoma. Changes in the regulations in 2014 allowed optometrists to initiate treatment including the most common family of ocular hypotensive agents, prostaglandin analogues, but required a referral to an ophthalmologist or ophthalmology service within four months. [31]. By contrast, ophthalmologists are medically trained and undergo an additional five years of training to attain specialist recognition in the Australian health system taking on leadership of glaucoma management, particularly for advanced cases. Optometry-led administration with ophthalmology oversight (through advising on the clinical service delivery model, and in the clinical board of management and stakeholder committees) was integral to this scheme’s success. This ensures regulatory compliance and suitable processes, such as the specific application of shared standardised referral forms, standardised electronic medical record forms and report templates, and the booking of follow up consultations at the conclusion of each patient attendance. Other enabling factors included the face-to-face co-delivery of protocol-based care in a neutral, community based clinical setting, without any on-site spectacle or other device sales, as well as access to ophthalmology supervision and expertise either in person or remotely at any time. This final point on the value of virtual clinical oversight has been emphasised previously [1,32]. It increases outpatient capacity, referral rates, and overall patient satisfaction [33,34] and might in the future evolve into a virtual process applying big data for a more robust evaluation of patient outcomes [35].

### Limitations

This study was limited by its retrospective, observational nature and failed to address patient satisfaction, false negative cases, medication adherence, adverse events (including comorbidities) and feasibility in another setting. Consistent with other shared care models in the field [3], initial implementation also did not take into consideration patient or carer experiences. Similarly, patient selection and entry criteria into the glaucoma management clinic were not included but have been described elsewhere [10]. A cost effectiveness analysis was also beyond the scope of this work though will evolve over time and likely depend on the equipment costs, patient-related factors such as adherence, treatment type, the statistically significantly increase in the number of visits for patients undergoing shared care (i.e. the distinctly shorter monitoring intervals), the rate of virtual and face-to-face re-referral (similar or lower than other schemes in the field) and the lack of optical appliance sales [3,36].

## Conclusion

In conclusion, this work uses longitudinal, three-year outcomes data to highlight the value of a virtual-review facilitated, hybrid shared care scheme in delivering effective and timely ongoing care for patients with chronic stable glaucoma at low risk of visual loss. The burden of work generated by identifying cases to be monitored in the service (460% increase in appointments), was almost equally shared between optometry and ophthalmology. The scheme was unique in allowing scope for safe, independent clinical decision making by optometrists, built off a decade long partnership between university-affiliated optometrists and the local public hospital ophthalmology department.

## Additional File

The additional file for this article can be found as follows:

10.5334/ijic.5470.s1 Supporting Information. Additional details on the study organisation.
