# Supplementary material for: Glaucoma Community Care: Does Ongoing Shared Care Work?
Source: Int J Integr Care. 2020 Aug 7;20(3):5. doi: 10.5334/ijic.5470 (PMC7427692; doi:10.5334/ijic.5470)
Supplement: Supporting Information. — Additional details on the study organisation. [file ijic-20-3-5470-s1.pdf]

Supporting Information

Supplementary File 1. Additional details on the study organisation

Patients are referred into the Centre for Eye Health (CFEH) Glaucoma management clinic (GMC) either internally or externally for a baseline assessment. All referrals are received using a standardised referral form and pre-screened by a CFEH optometrist. An initial assessment is then booked and conducted using a team approach, involving 1) an ophthalmic assistant, 2) a highly trained, therapeutically endorsed CFEH staff optometrist competent in performing gonioscopy and 3) a consultant ophthalmologist from the affiliated public hospital eye clinic. All patients seen in the GMC are assessed in accordance with pre-determined, standardised testing and reporting protocols. On presentation, all patients complete an entering questionnaire, which addresses the key components of the ocular and medical histories, including risk factors for glaucoma – age, ethnicity, family history, steroid use, migraine, peripheral vasospasm, eye injury, and systemic hypertension.[1] The patient's refraction is supplied by the referring practitioner. Mandatory baseline tests include: visual acuity, visual fields (Humphrey 24-2 SITA standard perimetry, Carl Zeiss Meditec, Dublin, California, USA), applanation tonometry, central corneal pachymetry (Pachmate pachymetry, DGH Technology Inc., Exton, PA USA), stereoscopic optic disc photography (Kowa WX-3D, Kowa Nagoya, Aichi, Japan), and optical coherence tomography (Cirrus HD-OCT, Carl Zeiss Meditec). In all cases, the ophthalmologist reviews the clinical history, performs slit lamp biomicroscopy, gonioscopy, funduscopy, reviews the test results and then confers with the optometrist on the diagnosis and treatment plan. The consensus diagnosis and management plan are then discussed and implemented with the patient and a report summarising all findings is prepared by the examining optometrist and transmitted to necessary parties (such as the referrer and/or patient's general practitioner). All follow up appointments are booked at the conclusion of each consultation irrespective of the appointment type and confirmed by client services staff the day prior to assessment.

Supplementary File 2. Coding protocol of the study

- The diagnosis of glaucoma was defined according to the reported consensus opinion of both the GMC examining optometrist and ophthalmologist, and categorised by study authors, EW and AL, into one of six categories: suspect, ocular hypertension (OHT), primary open angle glaucoma (POAG), normal tension glaucoma (NTG), secondary open angle glaucoma (SOAG) and other.
- Glaucoma severity was similarly extracted and coded into four groups: suspects, early, moderate or advanced based on the clinical diagnosis and visual fields results.[2 3] A visual field MD better than -6dB, between -6 and -12 dB, and worse than -12dB, was categorised as early, moderate or severe, respectively.[3] Visual field loss within 10 degrees of fixation[3] formed an additional criterion for advanced field loss and was defined as three or more points with an abnormal probability score of less than 2% across the twelve points falling within the central 10 degrees of the Humphrey 24-2 Visual Field Analyser 24-2 test grid.
- Other patient demographic and clinical data included: age, sex, ethnicity, refractive error, date of the consultation, total number of visits, visit type (in the ophthalmology-led GMC, optometry-led clinic or for a short subsequent consultation), IOP, central corneal thickness (CCT), visual field MD, management plan (start treatment, change treatment, continue treatment, stop treatment or no treatment), treatment type and recommended review period.

Table 1. Definitions used by the study coders

Element	Categories and definitions
Diagnosis	• Suspect: Patients with IOP within normal limits and no evidence of glaucomatous structural or functional damage<br>• Ocular hypertension (OHT): Patients with raised IOP and no evidence of glaucomatous structural or functional damage<br>• Primary open angle glaucoma (POAG): IOP >21mmHg<br>• Normal tension glaucoma (NTG): Patients showing structural and/or functional signs of glaucoma and an IOP ≤ 21mmHg in both eyes; this group may also include patients on previous treatment where the pre-treatment IOP was unknown.<br>• Secondary open angle glaucoma (SOAG): Including pseudoexfoliation or pigment dispersion syndrome<br>• Other: Includes congenital glaucoma and chronic angle closure glaucoma subtypes
Management plan	Change in management<br>• Change treatment<br>o Includes patients not reaching target using topical therapy and subsequently co-managed with the local hospital department using selective laser trabeculoplasty (SLT) or other laser treatment instead:<br>*"In light of the signs of progression and insufficient IOP reduction, we have discussed further treatment options with …. She has elected to undergo SLT instead of pharmacological treatment."*<br>o Includes patients with a former history of SLT who were initiated on monotherapy<br>*"They had SLT performed previously on OS only… [the patient] was commenced on Timoptol XE 0.25% OS only to be used each morning."*<br>o Includes patients switching from one form of monotherapy to another (within or outside of the same drug class)<br>*"We have switched [the patient] from Xalatan to Travatan OD"*<br>o Also includes cases transitioned from monotherapy to combination therapy or one combination therapy drug to another<br>*"…non-compliance with the Lumigan eyedrops… given the field progression in her right eye, we have commenced … on Ganfort PF once a night in both eyes."*<br>*"[The patient] has been advised to change his treatment from Xalacom nocte to Ganfort PF nocte OU."*<br>o Patients formerly on monocular treatment only, transitioned to binocular therapy<br>*"[The patient] has open angle glaucoma and is currently managed with DuoTrav OS only… [They have been] advised to use DuoTrav once in the morning in both eyes due to the possible field progression OD"*<br>o Includes patients on multiple drops (combination therapy) where one prescription was discontinued<br>*"… has been advised to continue with their current glaucoma treatment (Ganfort OU). She was advised that she did not need to take timolol."*<br>o Dosing change<br>*"[The patient] has been advised to continue with their current glaucoma treatment and to instil the eyedrops in the morning."*<br>• Start treatment<br>o Initiation of glaucoma related treatment either at a patient's baseline visit but may have also occurred during subsequent consultations<br>• Stop treatment<br>o Describes a change in management where topical therapy for glaucoma is terminated e.g. at the conclusion of a treatment trial or due to a change in risk status<br>No change in management<br>• No treatment<br>• Continue treatment

Abbreviations: IOP, intraocular pressure

References

1. Australian Government National Health and Medical Research Council. NHMRC Guidelines for the Screening, Prognosis, Diagnosis, Management and Prevention of Glaucoma, 2010.
2. Kim JM, Kyung H, Shim SH, et al. Location of Initial Visual Field Defects in Glaucoma and Their Modes of Deterioration. Investigative ophthalmology & visual science 2015;**56**(13):7956-62.
3. RANZCO. Guidelines for Collaborative Care of Glaucoma Patients, April 2015.
